# Hybrid origin of European commercial pigs examined by an in-depth haplotype analysis on chromosome 1

**DOI:** 10.3389/fgene.2014.00442

**Published:** 2015-01-05

**Authors:** Mirte Bosse, Ole Madsen, Hendrik-Jan Megens, Laurent A. F. Frantz, Yogesh Paudel, Richard P. M. A. Crooijmans, Martien A. M. Groenen

**Affiliations:** Animal Breeding and Genomics Centre, Wageningen UniversityWageningen, Netherlands

**Keywords:** *Sus scrofa*, hybridization, domestication, introgression, genetic variation, haplotype homozygosity

## Abstract

Although all farm animals have an original source of domestication, a large variety of modern breeds exist that are phenotypically highly distinct from the ancestral wild population. This phenomenon can be the result of artificial selection or gene flow from other sources into the domesticated population. The Eurasian wild boar (*Sus scrofa*) has been domesticated at least twice in two geographically distinct regions during the Neolithic revolution when hunting shifted to farming. Prior to the establishment of the commercial European pig breeds we know today, some 200 years ago Chinese pigs were imported into Europe to improve local European pigs. Commercial European domesticated pigs are genetically more diverse than European wild boars, although historically the latter represents the source population for domestication. In this study we examine the cause of the higher diversity within the genomes of European commercial pigs compared to their wild ancestors by testing two different hypotheses. In the first hypothesis we consider that European commercial pigs are a mix of different European wild populations as a result of movement throughout Europe, hereby acquiring haplotypes from all over the European continent. As an alternative hypothesis, we examine whether the introgression of Asian haplotypes into European breeds during the Industrial Revolution caused the observed increase in diversity. By using re-sequence data for chromosome 1 of 136 pigs and wild boars, we show that an Asian introgression of about 20% into the genome of European commercial pigs explains the majority of the increase in genetic diversity. These findings confirm that the Asian hybridization, that was used to improve production traits of local breeds, left its signature in the genome of the commercial pigs we know today.

## Introduction

Domestication is a complex process that has major implications for both phenotypic and genetic variation. It is not an exception that the domesticated form appears to be very different from the wild species in terms of phenotype and genetic makeup. Examples include multiple crop species (Doebley et al., 2006), dogs (vonHoldt et al., 2010) and farm animals (Andersson, 2001; Dobney and Larson, 2006). The differences are caused mainly by two phenomena: (1) selection for particular traits in the domesticated population including domestication genes, which can either facilitate the maintenance of the species in question or have commercial interest; (2) hybridization with individuals from highly divergent populations to improve selected traits. The domesticated pig (*Sus scrofa*) is a good example of such a species, since the domesticated form, as well as its wild relatives, is widespread across the Eurasian continent although phenotypically it can be highly distinct. Domestication of the pig is known to have its origin independently in the Near East and in Asia roughly 10,000 years ago (ya), which led to at least two distinct domestication clades (Kijas and Andersson, 2001; Larson et al., 2005).

Strong artificial selection after the initial domestication led to a wide variety of breeds, each with distinct phenotypes, and selective signatures in the genome (Rubin et al., 2012; Wilkinson et al., 2013). Breed formation and artificial selection for particular traits can drastically reduce genetic diversity, which has been shown for multiple species (Kristensen and Sorensen, 2005; Taberlet et al., 2008). Surprisingly, in pigs, the commercial breeds in Europe are generally more diverse than their wild counterparts (Groenen et al., 2012; Bosse et al., 2014a). In this research we examine which process contributed most to the difference in genetic diversity between European commercial breeds and European wild boars.

In Europe, pig domestication did not occur as a single, unique event, but rather was a continuous process of domestication, isolation and hybridization that led to the domestic European pigs seen today (Larson et al., 2007). Furthermore, glaciations likely had a major impact on the genetic diversity in European wild boar (Scandura et al., 2008). It has been suggested that there were multiple refugia in Europe during the last glaciation, resulting in many private haplotypes for the separate populations (Alves et al., 2010). In the drawn-out process of domestication of the pig in Europe, the mixing of wild boar genetic variation from different regions in Europe, might explain the high diversity found in modern European pigs. Although variation has been lost locally in most European wild populations, the combined genetic diversity from geographically isolated populations should display similar patterns of genetic diversification as is shown for European commercial haplotypes. The first hypothesis we test, therefore, is that the European breeds are a combination of separate European populations that have been amalgamated into a single population, resulting in higher levels of variation.

Introgression from Asian pigs into European breeds was first demonstrated with molecular data by Giuffra et al. (2000), and indeed multiple international breeds have subsequently been found to contain Far Eastern mitochondrial haplotypes (Clop et al., 2004; Fang and Andersson, 2006). Ramirez et al. (2009) suggested that this introgression was mostly female driven, because of the predominance of the European HY1 Y-chromosomal haplotype in European domestic pigs. An Asian origin for multiple commercially important phenotypes has been shown to be the result of this hybridization (Ojeda et al., 2008; Wilkinson et al., 2013; Bosse et al., 2014b; Hidalgo et al., 2014). Alves et al. (2003) showed that not all European local domestic breeds, such as Iberian pigs, contain mtDNA of Asian origin, and based on studies of genomic DNA, varying levels of admixture in local breeds have been suggested (Herrero-Medrano et al., 2013). We recently found that regions in the genome of Large White pigs that contain DNA that is shared with Asian pigs are generally more diverse than regions that do not share DNA with Asian haplotypes (Bosse et al., 2014a). However, it is unknown whether this is a direct result of the introgression (rather than, for example, incomplete lineage sorting). Moreover, how much the introgressed Asian haplotypes contributed to variation in the genome of European commercial pigs remains an unanswered question. Thus, the second hypothesis we test is that the Asian introgression has led to higher diversity in the European commercial pigs.

For prioritizing farm animal genetic resources (FanGR) for conservation, it is important to know the distribution and the origin of variation in the (domesticated) species (Groeneveld et al., 2010). With this work, we make a contribution by analyzing the details of genetic diversity on chromosome 1 within and between groups of pigs and wild boars in Asia and Europe.

## Materials and methods

### Data

The data used for this paper consists of all variants on chromosomes 1, 2, and 18 that were observed in 136 pigs. These variants have previously been deposited into dbSNP (release 138). The data was obtained by aligning Illumina paired-end 100bp reads to the *Sus scrofa* reference genome (build 10.2) using Mosaik Aligner (V.1.1.0017). Reads were trimmed to a minimum base PHRED quality of 20 averaged over 3 consecutive bases and only mate pairs with both reads at least 45 bp in length were included. Each individual was sequenced to ~10× depth of coverage. SNPs were called separately per individual with SAMtools (V. 0.1.13) pileup with a minimum coverage of 4x and with at least 2 reads supporting the alternative allele. Sites were filtered for a minimum genotype and mapping PHRED quality of 20. Most of our analyses were based on all 2,747,210 variants called on chromosome 1. From the original matrix containing all variable sites in all 136 pigs, indels were excluded and SNP loci were retained if called in >80% of all individuals. The minimum coverage of genotypes called within each group of pigs was set to >80%, resulting in 410,237 high-quality SNPs on chromosome 1. All individuals were imputed and phased for these 410,237 SNPs with Beagle v.3.3.2. Although it is unknown whether the two haplotypes represent the actual phases, we considered them to be one full-length haplotype, as uncertainties in phase should balance out when homozygosity rates are calculated for all haplotype pairs in the dataset. We pooled the haplotypes from pigs belonging to the 8 groups listed in Table 1.

**Table 1:** **Number and haplotypes per group and background of sequenced individuals**.

**Group**	**No. haplotypes**	**Codes**	**Population details**
Outgroup	4	INDO	(wild) Sumatran *Sus scrofa*
European local	32	AS,BB,BK,BS,GO,LB,LE,LS,MW,TA,NS	Heritage breeds (Old British breeds), Less global breeds (Linderodsvin, Bunte Bentheimer, Angler Sattelschwein, Leicoma, Nera Siciliana)
European Iberian	22	CA,CM,CS,CT, MA,NI	Pigs from the Iberian peninsula (Mangalica, Negro Iberico, Casertana, Chato Murciano, Calabrese, Cinta Senese)
European commercial	94	DU,HA,LR,LW,PI	Widespread commercial breeds (Duroc, Hampshire, Landrace, Large White, Pietrain)
European wild	52	WB21,22,25,26,28,31,32,33,42,44,72	Wild boar from Western, South-Eastern and Southern Europe (Netherlands, France, Spain, Italy, Switzerland, Greece, Samos, Armenia)
Asian commercial	30	JQ,MS,XI	Asian breeds known to be commercially important (Meishan, Xiang, Jianquahai)
Asian local	18	JI,LSP,TH,WS,ZA	Local breeds and wild pigs (Jinhua, Leping spotted, Wannan spotted, Zhang, Thai)
Asian wild	20	WB20,29,30	North China, South China, Japan

**Figure 1:**
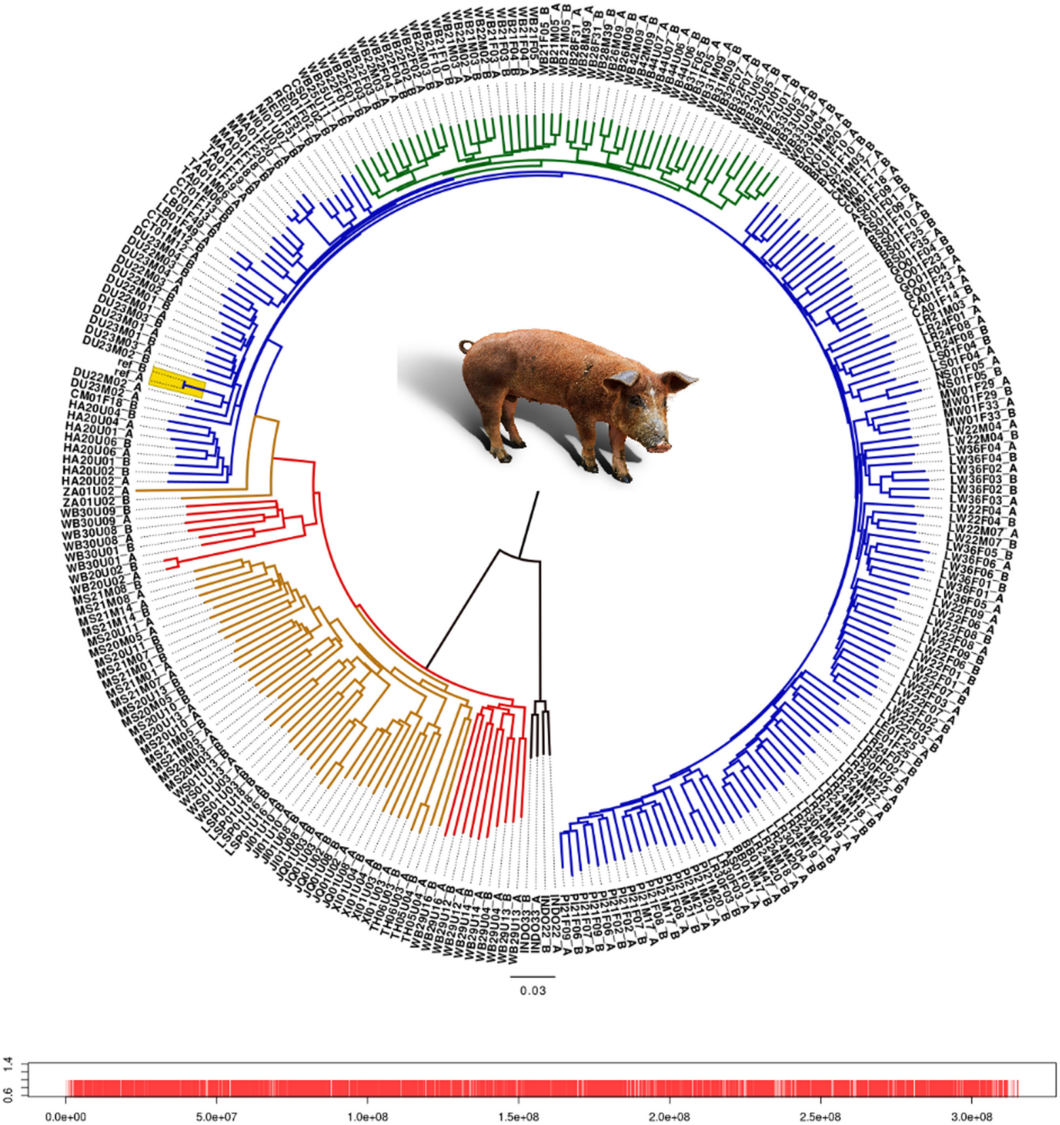
**Neighbor-joining tree of all haplotypes of chromosome 1**. Each individual has 2 haplotypes, one labeled after the name of the individual with the suffix “A” and the second haplotype contains the suffix “B.” Red line, Asian wild haplotype; orange line, Asian commercial or local haplotype; blue line, European commercial or local haplotype; green line, European wild haplotype. Locations of the markers on chromosome 1 are indicated by red bars. Alleles from the pig reference genome are included as two separate haplotypes without variation between them, and are highlighted in yellow.

The group name of the pigs under “group” is how this group of individuals is referred to in the rest of the text. The codes of all pigs correspond to their labels in **Figure 1**. The details of the populations or breeds that the pigs belong to are summarized in the column “Population details.” Note that information for the European local and Asian local individuals can be limited, and therefore these are rather heterogeneous groups.

### Phylogenetic analysis

To assess the relationship of haplotypes in our dataset, we constructed a phylogenetic tree based on the phased haplotypes. Each haplotype was considered as an independent sample, so that haplotypes belonging to the same individual do not necessarily need to cluster together. Because missing sites were imputed with Beagle, no missing alleles were present in the phased haplotypes. Sites with more than two alleles were removed from the data and a distance matrix was constructed in PLINK (Purcell et al., 2007). NEIGHBOR (PHYLIP V. 3.695; Felsenstein, 2005) was used to build a neighbor-joining tree for all haplotypes using two Sumatran *Sus scrofa* as outgroup, and the tree was depicted using FIGTREE (http://tree.bio.ed.ac.uk/software/figtree/).

### Haplotype homozygosity analysis

#### Analysis 1

After individuals were phased for the full length of chromosome 1, the homozygosity was analyzed between two haplotypes spanning the full chromosome for all possible combinations of two haplotypes in the dataset. Haplotype homozygosity is defined as the proportion of homozygous sites between two paired haplotypes, and ranged from 0 to 1. We calculated haplotype homozygosity as the proportion of all sites (410,237) that occurred in homozygous state, so that 0 represents only heterozygous loci and 1 represents complete homozygosity between both haplotypes. We then paired all possible combinations of two haplotypes in the dataset and determined the homozygosity of these hypothetical diploid individuals in R (see Box 1). Haplotype homozygosity was pooled for pairs of haplotypes belonging to the same group (Table 1), so that we ended up with a distribution of homozygosity within a group that represents the full range of variation between haplotypes in a group. Within-group haplotype homozygosity was then compared between the different groups. In the second part of this analysis haplotypes from two different groups were paired and the haplotype homozygosity for these mixed pairs was computed to obtain a distribution of homozogosities between haplotypes. This distribution was then compared with the distribution of homozygosity between haplotypes from two other groups.

Box 1 **Principles of the analyses.**

**Figure:**
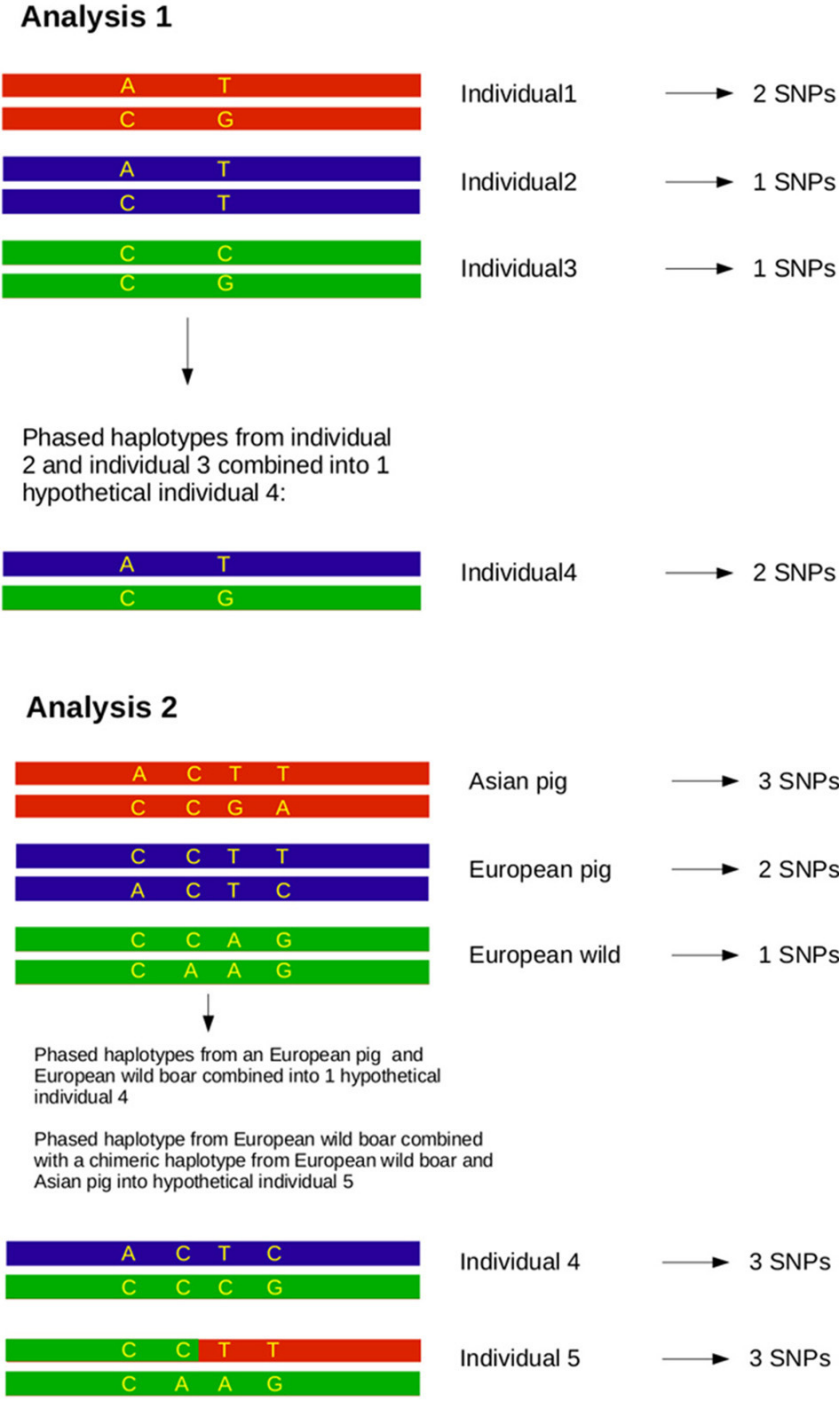

#### Analysis 2

Previous estimates on the fraction of Asian DNA ranged from 20 to a maximum of 35% (Groenen et al., 2012; Bosse et al., 2014b). In the second analysis we wanted to assess the influence of Asian introgression into a European haplotype. In order to do this, we simulated introgression by transferring 15, 20, and 25% of a haplotype belonging to the Asian commercial group into a haplotype that belongs to the European wild group (see Box 1). We used a custom perl script to construct these chimeric haplotypes in which 15, 20, or 25% of the alleles coming from an Asian commercial haplotype replace the alleles in a European wild haplotype. All possible pairs between European wild and Asian commercial haplotypes to construct a chimeric haplotype were included. Then, these chimeric haplotypes were again paired with all possible European wild haplotypes (except for the one that the chimeric haplotype is constructed of) and the homozygosity between the two haplotypes was calculated as described for analysis 1. These haplotype homozygosities were pooled so that a distribution of haplotype homozygosity in the artificially created Asian-European hybrids was obtained.

### Consistency over chromosomes

All analyses presented in this paper are based on haplotypes spanning the full length of chromosome 1. We selected this chromosome because it is the longest pig chromosome and therefore the introgression signals are probably most representative for the full genome and less prone to occasional aberrations due to a limited recombination/drift. However, to check whether chromosome 1 is representative for the complete genome, we compared the haplotype homozygosities for the same pairs of individuals between chromosome 1 and two other chromosomes: chromosome 2 (the second longest chromosome), and the shortest and acrocentric chromosome 18. We tested the correlation coefficient between the haplotype homozygosities of the different chromosomes with Pearson's product-moment correlation in R.

### Runs of homozygosity

We extracted runs of homozygosity (ROH) from all combinations of paired haplotypes coming from the European pigs and wild boars. ROHs were called with the –homozyg option using PLINK v1.07, allowing for one heterozygous site in the ROH and a minimum ROH size of 10Kb.

## Results and discussion

### Variation within groups

We analyzed the phylogenetic relationship of all haplotypes spanning chromosome 1 by constructing a neighbor-joining tree (Figure 1). The Asian and European haplotypes form two distinct clusters, which is consistent with the hypothesis of independent domestication (Kijas and Andersson, 2001; Larson et al., 2005; Groenen et al., 2012; Ramírez et al., 2014). European wild boars constitute a monophyletic clade within the European commercial pigs. The pig reference genome sequence (Groenen et al., 2012) clusters within a group of Duroc pigs, which is expected because the reference genome is based on a female Duroc. The Chinese commercial and local haplotypes cluster with the Northern and Southern Chinese wild haplotypes. The only exception is the Zhang pig, which is closer to European pigs (labeled “ZA” in Figure 1). This individual is possibly introgressed with European breeds and therefore we mention explicitly when this individual is included in the analysis. Haplotypes from the same individual generally cluster together, but within the European commercial group this is not always the case, showing the close relationship of these individuals. The Japanese wild boar (WB20) and the Mangalica pigs (MA) are the most inbred individuals, with homozygosity between the two haplotypes within each individual above 0.99.

**Figure 2:**
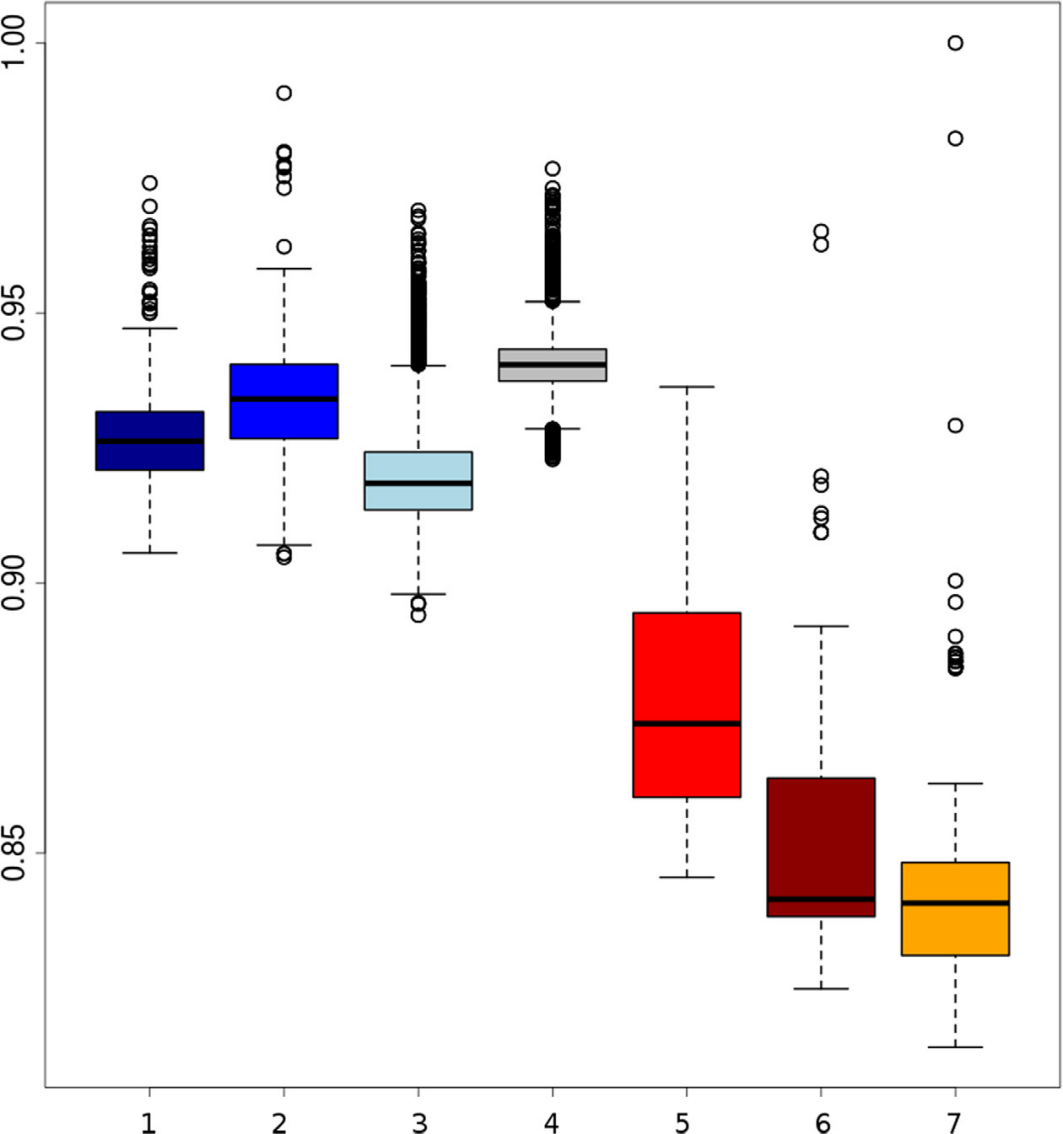
**Boxplots of homozygosity between two randomly paired haplotypes within groups**. (1) Darkblue, two European local haplotypes; (2) blue, two European Iberian haplotypes; (3) lightblue, two European commercial haplotypes; (4) gray, two European wild haplotypes; (5) red, Asian commercial haplotypes; (6) brown, Asian local haplotypes; (7) orange, Asian wild haplotpyes (the highest dot indicates haplotype homozygosity within the Japanese wild boar).

Branches within the Asian cluster are longer than those for European haplotypes. When the homozygosity between two haplotypes from individuals with the same background is measured, the variation (within groups) between two Asian haplotypes is indeed higher than between two European haplotypes from the same group, except for the Japanese wild boar (Figure 2). This is congruent with previous findings that *Sus scrofa* has its origin in Asia (Groenen et al., 2012; Frantz et al., 2013) and that European pigs experienced a stronger bottleneck during the last glaciation, resulting in reduced variation (Bosse et al., 2012). Independent domestication should lead to Asian local and commercial pigs being more variable than European pigs, which has been shown previously based on microsatellite data (Megens et al., 2008) and sequence data (Bosse et al., 2012) and is also supported by our analysis (Figure 2).

Before and even after the establishments of modern breeds, hybridization between different European populations was common practice. Therefore European commercial pigs are all thought to contain Asian haplotypes. However, this is not necessarily the case for all local breeds in Europe. Our results show that variation between haplotypes from European local breeds is lower than between European commercial haplotypes, which could be due to less Asian introgression or because they have a less mixed European origin (Herrero-Medrano et al., 2013). Some breeds from the Iberian peninsula and old British heritage breeds cluster with the European wild boar (Figure 1) which suggests that the source population for domestication more closely resembles these breeds and wild boar, and that genetic differentiation between those pigs is low as recently described by Ramírez et al. (2014). In line with our expectations, we find that variation between two European wild haplotypes is generally lower than between two European commercial haplotypes, especially when variation within individuals is not considered. These findings serve as initial concept of our further analyses.

### Consistency over chromosomes

We did an in-depth analysis of haplotypes on chromosome 1, but first verified whether chromosome 1 is actually a representative model for the rest of the (autosomal) genome. The correlation between haplotype homozygosity for pairs of haplotypes of chromosome 1 and haplotype homozygosity for chromosome 18 is 0.9848, and between chromosome 1 and chromosome 2 is 0.9874. Looking at the homozygosities for pairs of haplotypes on chromosome 1 and pairs of haplotypes on chromosome 18 (Figure S1), two small clouds of dots stand out: one having a higher homozygosity on chromosome 18 (red) and the other having a lower homozygosity on chromosome 18 compared to chromosome 1 (orange). These clouds actually represent the haplotypes from only two Asian pigs WS01U03 (red) and ZA01U02 (orange) in combination with all European haplotypes, suggesting a different level of European introgression into the different chromosomes for these two pigs. Since the overall correlation coefficients are so high for the rest of the paired haplotypes in the dataset, we conducted the rest of the analyses only on chromosome 1 and excluded these two individuals from further analyses.

### Variation in wild boars

**Figure 3:**
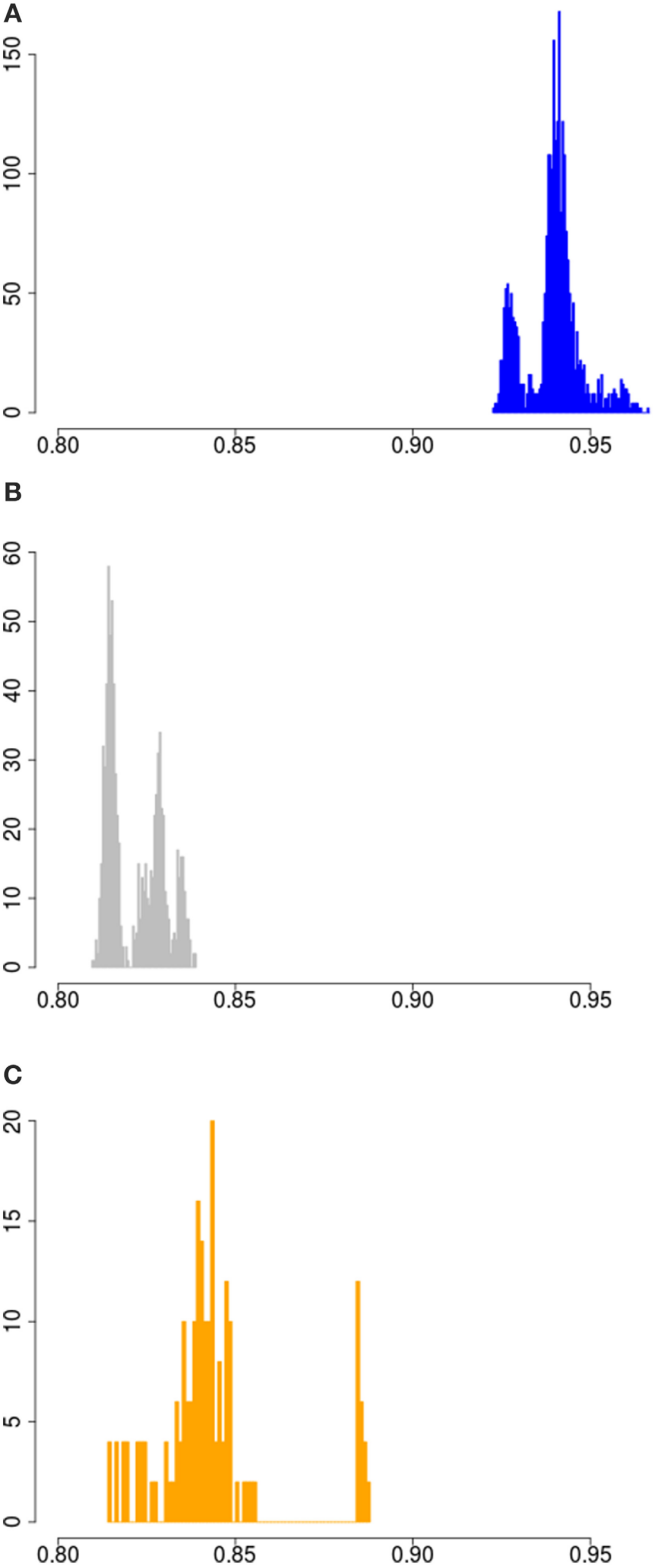
**Homozygosity between paired wild haplotypes**. **(A)** Haplotype homozygosity between all possible pairs of European wild haplotypes. **(B)** Haplotype homozygosity between all possible pairs of 1 European wild and 1 Asian wild haplotye. **(C)** Haplotype homozygosity between all possible pairs of two Asian wild haplotypes.

*Sus scrofa* probably originated in South-East Asia. To assess the full width of variation that is present within the species in the wild, we measured variation for all possible pairs of haplotypes in the dataset. The lowest homozygosity between haplotypes is observed when a haplotype is paired with an outgroup haplotype (the peak at ~0.72 in Figure S1). The geographic region closest to the center of origin is often the richest in genetic diversity, as shown for other species like dogs and humans (Long and Kittles, 2003; vonHoldt et al., 2010). Indeed, our analysis corroborate that the divergence between haplotypes is larger when at least one haplotype is Asian than when no Asian haplotypes are present (Figures 2, 3). Eastern and Western *Sus scrofa* diverged around 1.2 Mya and this divergence resulted in a multitude of fixed differences between both wild populations (Groenen et al., 2012). Naturally, this divergence also contributes to genetic variation within the species, and to quantify the unique contributions of both continents to variation within the species we looked at the difference in homozygosity between paired haplotypes from the same continent and paired haplotypes from Europe and Asia. For mainland *Sus scrofa*, most divergence between haplotypes is found when a European wild haplotype is pooled with an Asian haplotype, regardless its domestication status. The fact that we do not find a significant difference in homozygosity between an Asian wild or Asian local and commercial haplotype paired with a European wild haplotype suggests that the time since the most recent common ancestor is similar and that generally no or very little introgression from Europe into our sampled Asian domesticated breeds has occurred. The homozygosity of European wild haplotypes paired with Asian wild is lower than that of two Asian wild haplotypes (averages of 0.825 and 0.84, Figure 3), but the difference is far less pronounced than the difference in homozygosity between two European wild haplotypes and the mixture between European and Asian (0.94 vs. 0.825, Figure 3). This indicates that the largest source of variation comes from the Asian wild boars, and that despite the ~1.2 My divergence between European and Asian populations, the European clade contributes marginally to the genetic diversity of the species as a whole. The finding that populations further away from the source population capture less genetic diversity is consistent with other species.

### Variation between european haplotypes

**Figure 4:**
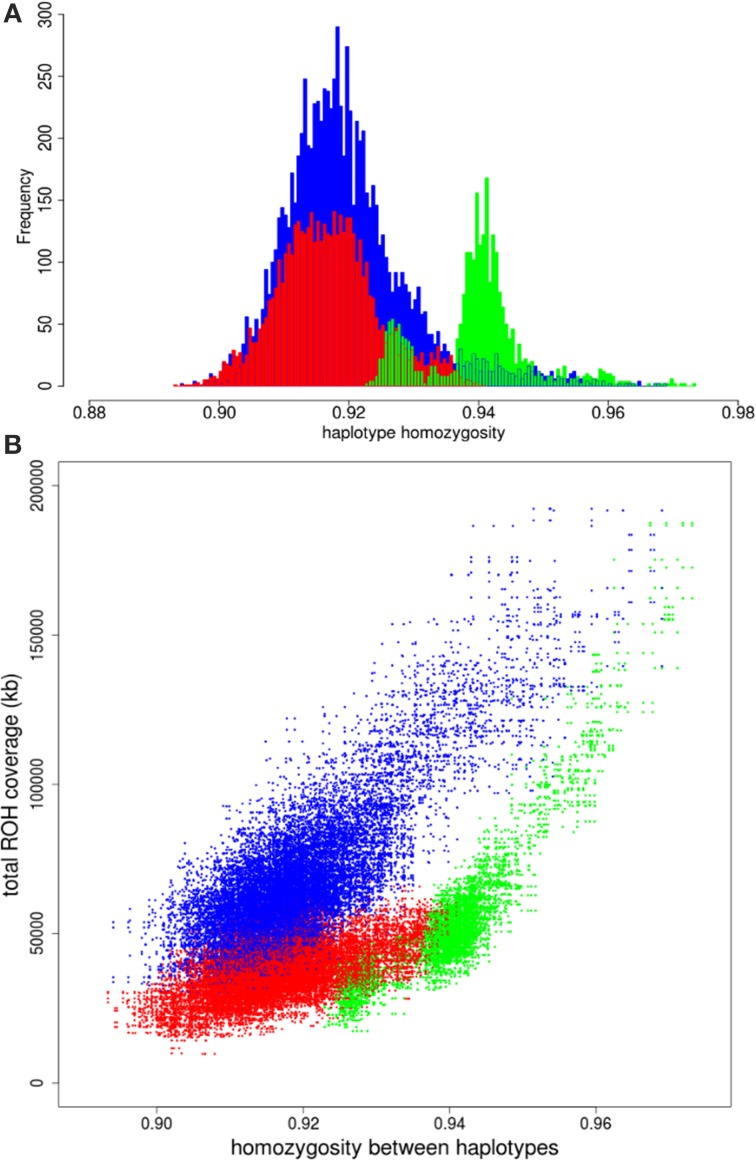
**Homozygosity between paired haplotypes in Europe**. **(A)** Homozygosity between two European wild haplotypes is displayed in green. Homozygosity between two European commercial haplotypes is in red and the blue bars indicate homozygosity between one European wild and one European domesticated haplotype. **(B)** Homozygosity between haplotypes over the full chromosome on the x-axis is plotted against total ROH coverage between haplotypes on the y-axis for three combinations: two European commercial haplotypes (blue); two European wild haplotypes (green); one European wild and one European commercial haplotype (red).

We had a closer look at the cause of the difference in variation within Europe. One of our hypothesis was that if the higher variation in the commercial lines is mainly caused by a mixture of different European populations, the distribution of variation between two European haplotypes should overlap with the distribution of variation between European commercial haplotypes. The European wild boars used in the current study are derived from different glaciation refugial origins and should therefore represent well extant wild boar variation throughout Europe. All possible pairs of haplotypes from European wild origin should therefore result in a distribution that exceeds the lowest haplotype homozygosity of all pairs of European commercial haplotypes, because the most divergent haplotypes from Europe are included in the European wild distribution. The far tail of the distribution of European wild haplotypes with most variation does not even overlap the mean of variation between two commercial European haplotypes (Figure 4A), indicating that two wild European haplotypes show more homozygosity than two random European commercial haplotypes, even if these wild haplotypes are sampled from very divergent populations. This suggests that the variation within the European commercial group cannot be completely explained by a mixture of European wild haplotypes. Therefore, it is highly unlikely that the relatively high degree of variation (compared to European wild boar) that is generally found within the European commercial breeds, is due to a mixture of European wild haplotypes, as assumed in hypothesis 1. The distributions for paired haplotypes within the European local and European Iberian group have lower means than the European wild group as well, and their extremes also exceed the European wild distribution. These findings suggest that even some local breeds may contain introgressed haplotypes.

#### Runs of homozygosity (ROH)

Another possibility of the higher variation in European commercial breeds is that European wild boar populations experienced strong recent bottlenecks and associated loss of diversity after the split with European domestic pigs (domestication). We compared the correlation between total ROH coverage on chromosome 1 (as inferred from PLINK) and homozygosity between haplotypes for the European commercial breeds and European wild boar. ROHs between two commercial European haplotypes are slightly more abundant and longer than ROHs between one European commercial and one European wild haplotype (Figures S2A,B). By contrast, more ROHs are found between two European wild haplotypes than between a European wild and a European commercial haplotype (Figures S2C,D). The average length of the ROHs between two European wild haplotypes is generally the same as between a European wild and a European commercial haplotype, unless haplotypes belong to the same European wild population (e.g., within the Netherlands). If the higher level of homozygosity between European wild haplotypes would have been caused by recent inbreeding, the coverage of ROH on chromosome 1 should be higher between two European wild haplotypes than between two European commercial haplotypes. As can be seen in Figure 4B, the haplotype homozygosity between two European wild haplotypes is higher than between two European commercial haplotypes with the same level of ROH coverage. These findings suggest that recent inbreeding (i.e., the occurrence of ROH) does not explain the higher homozygosity between wild haplotypes compared to commercial haplotypes.

### The effect of introgression

#### Pairing with Asian haplotypes

**Figure 5:**
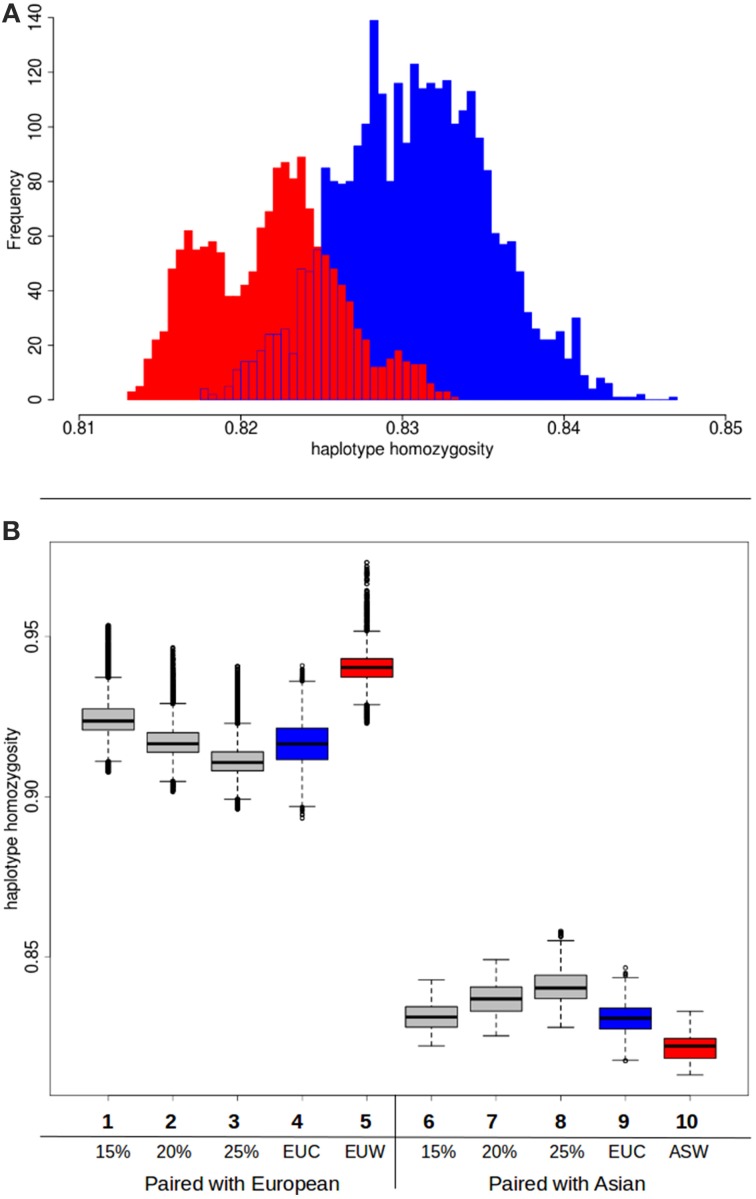
**Haplotype homozygosity with Asian introgression**. **(A)** Homozygosity between haplotypes when Asian commercial haplotypes are paired with European commercial (red) or European wild (blue). **(B)** Boxplots of haplotype homozygosity. Haplotypes are paired with European wild haplotypes (left) or Asian commercial haplotypes (right). Red boxes indicate haplotypes paired with European wild haplotypes. Blue boxes represent haplotypes that are paired with European commercial haplotypes. Gray boxes represent the distribution of homozygosity when the haplotype is paired with a chimeric haplotype that is a combination of a European wild haplotype and a Asian commercial haplotype (see also Box 1 in Supplementary material). (1) European wild paired with 15% Asian chimeric haplotype (2) European wild paired with 20% Asian chimeric haplotype (3) European wild paired with 25% Asian chimeric haplotype (4) European wild paired with European commercial (5) European wild paired with European wild (6) Asian commercial paired with 15% Asian chimeric haplotype (7) Asian commercial paired with 20% Asian chimeric haplotype (8) Asian commercial paired with 25% Asian chimeric haplotype (9) Asian commercial paired with European commercial (10) Asian commercial paired with European wild.

Although the hypothesis that different source populations in Europe caused the higher diversity in commercial pigs can be rejected based on these previous analyses, our second hypothesis, that Asian introgression caused the higher diversity, is not immediately confirmed. In a previous study (Bosse et al., 2014b) we showed that within the genome of a commercial European pig, the variation is higher when at least one Asian haplotype is present. This observation however does not confirm the role of Asian introgression either, since the presence of an Asian haplotype can be due to incomplete lineage sorting or recent introgression. Another potential cause of the increased variation is hybridization with an unknown population, so called “ghost admixture.” Introduced haplotypes from an unknown source are likely to increase variation in the European commercial population. Since this source should be unrelated to any of the pig groups here studied, pairing of a commercial European haplotype and an Asian haplotype should not result in less variation than an European wild haplotype paired with an Asian haplotype. If, however, the higher variation in European commercial genomes is due to Asian introgression, pairing with an Asian haplotype should result in higher homozygosity when a commercial European haplotype is used than when a wild European haplotype is used. We do find a small but significant difference between the European wild and European commercial haplotypes when they are paired with a commercial Asian haplotype (Figure 5A). As expected, the pairing with a European commercial haplotype results in less variation than the European wild haplotypes. Together with the lower haplotype homozygosity in the European commercial group, these findings indeed suggest that the introgression is Asian derived, or at least that the introgressed haplotypes are genetically more similar to Asian haplotypes.

#### Variation with chimeric haplotypes

In order to test whether the influx of Asian haplotypes caused the increase in homozygosity between the Asian wild and European commercial group, and to quantify this amount, we created composite haplotypes that contained 15, 20, and 25% of an Asian commercial breed haplotype and 85, 80 and 75% of a European wild haplotype as described in Box 1. These percentages were chosen because the introgression fraction from Asia into the European commercial pigs has previously been estimated to be between 15 and 35% (Fang and Andersson, 2006; Groenen et al., 2012; Bosse et al., 2014a). If the percentage of introgression is around 20%, then the distribution of haplotype homozygosity when a European commercial haplotype is paired with a European wild haplotype should strongly overlap the distribution when a composite haplotype containing 20% Asian commercial alleles is paired with a European wild haplotype. On top of that, the distribution of the chimeric haplotype paired with an Asian haplotype should overlap that of a European commercial haplotype paired with a Asian haplotype. The results show (Figure 5B) that pairing of a chimeric haplotype of European wild and Asian commercial with a European wild haplotype indeed results in a similar distribution of homozygosity as a pair between a European wild and a European commercial haplotype. Mean haplotype homozygosity shifts from 0.941 to 0.917, suggesting 20% introgression of Asian haplotypes. Our results confirm the previous estimates of around 20% admixture and demonstrate that the Asian introgression decreased haplotype homozygosity within Europe. In addition, we show that the haplotype homozygosity when a chimeric haplotype is paired with an Asian commercial haplotype increases compared to a European wild haplotype paired with an Asian commercial haplotype. The mean of the 15% Asian chimeric haplotypes is closest to the mean of a European commercial haplotype paired with an Asian commercial haplotype (Figure 5B), supporting the hypothesis that the introgression indeed comes from Asia.

## Conclusions

We confirmed Asia as the biggest source of genetic variation in *Sus scrofa*, in line with its geographical origin. The higher variation in the European commercial pigs compared to the European wild boar is largely explained by introgression of Asian haplotypes, rather than a mixture of European backgrounds.

### Conflict of interest statement

The authors declare that the research was conducted in the absence of any commercial or financial relationships that could be construed as a potential conflict of interest.

## Supplementary material

Figure S1 **Haplotype homozygosity and consistency over chromosomes. (A)** Distribution of haplotype homozygosity between all possible pairs of haplotypes on chromosome 1. The first peak around 0.725 contains all haplotypes paired with a haplotype from Sumatra. The second peak at 0.825 represents all haplotypes paired with a Chinese wild or Chinese commercial/local haplotype. The third peak round 0.92 shows all paired European haplotypes. **(B)** Consistency over chromosomes. The x-axis displays homozygosity between haplotypes from chromosome 1, and the y-axis shows the homozygosity between the same pairs of haplotypes for chromosome 18.

Figure S2 **Runs of homozygosity between paired haplotypes**. ROHs on chromosome 1 are recorded between pairs of haplotypes that belong to the European wild group (green) or the European commercial group (blue). **(A)** Number of ROH and average ROH length when a haplotype is paired with a European commercial haplotype. **(B)** Number of ROH and total ROH length when a haplotype is paired with a European commercial haplotype. **(C)** Number of ROH and average ROH length when a haplotype is paired with a European wild haplotype. **(D)** Number of ROH and total ROH length when a haplotype is paired with a European wild haplotype.
