# Supplementary figures and images for: Hybrid origin of European commercial pigs examined by an in-depth haplotype analysis on chromosome 1
Source: Front Genet. 2015 Jan 5;5:442. doi: 10.3389/fgene.2014.00442 (PMC4283659; doi:10.3389/fgene.2014.00442)

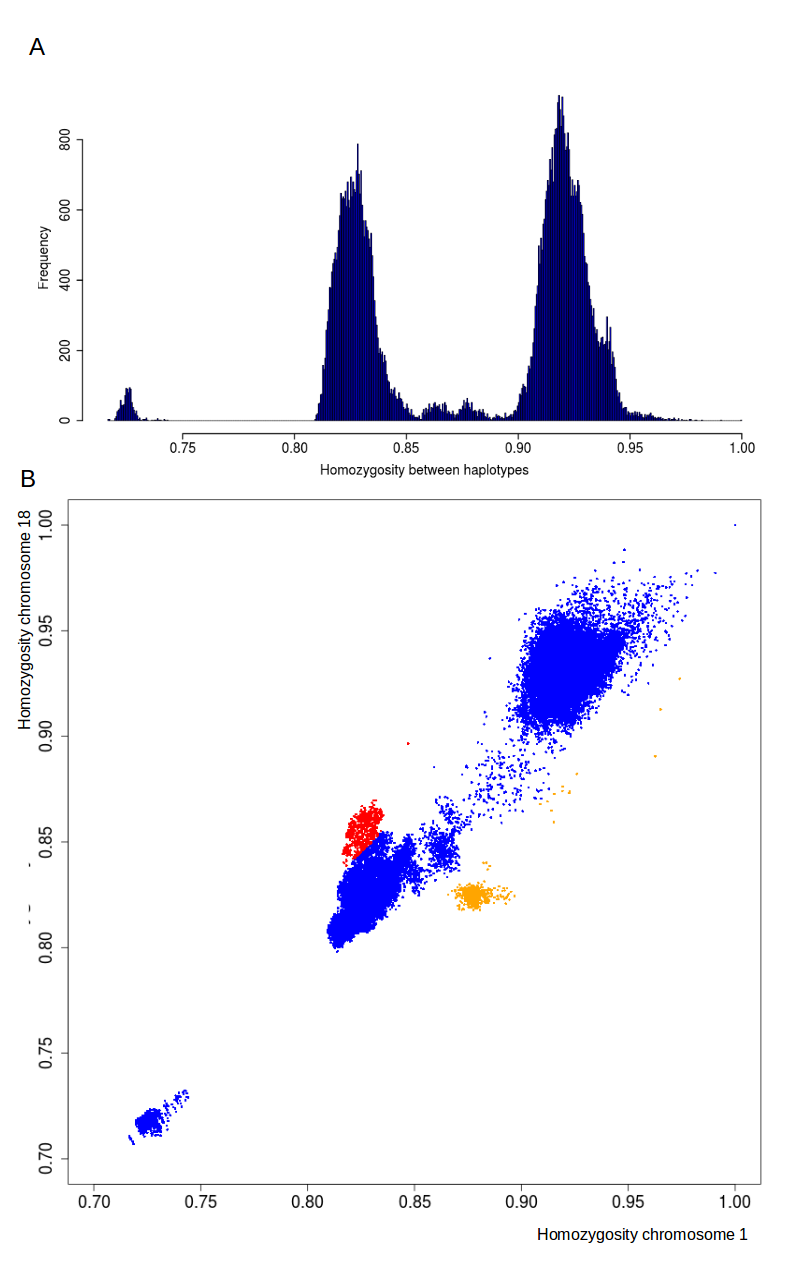

Supplement: Figure S1 — Haplotype homozygosity and consistency over chromosomes. (A) Distribution of haplotype homozygosity between all possible pairs of haplotypes on chromosome 1. The first peak around 0.725 contains all haplotypes paired with a haplotype from Sumatra. The second peak at 0.825 represents all haplotypes paired with a Chinese wild or Chinese commercial/local haplotype. The third peak round 0.92 shows all paired European haplotypes. (B) Consistency over chromosomes. The x-axis displays homozygosity between haplotypes from chromosome 1, and the y-axis shows the homozygosity between the same pairs of haplotypes for chromosome 18. [file Image1.TIF]

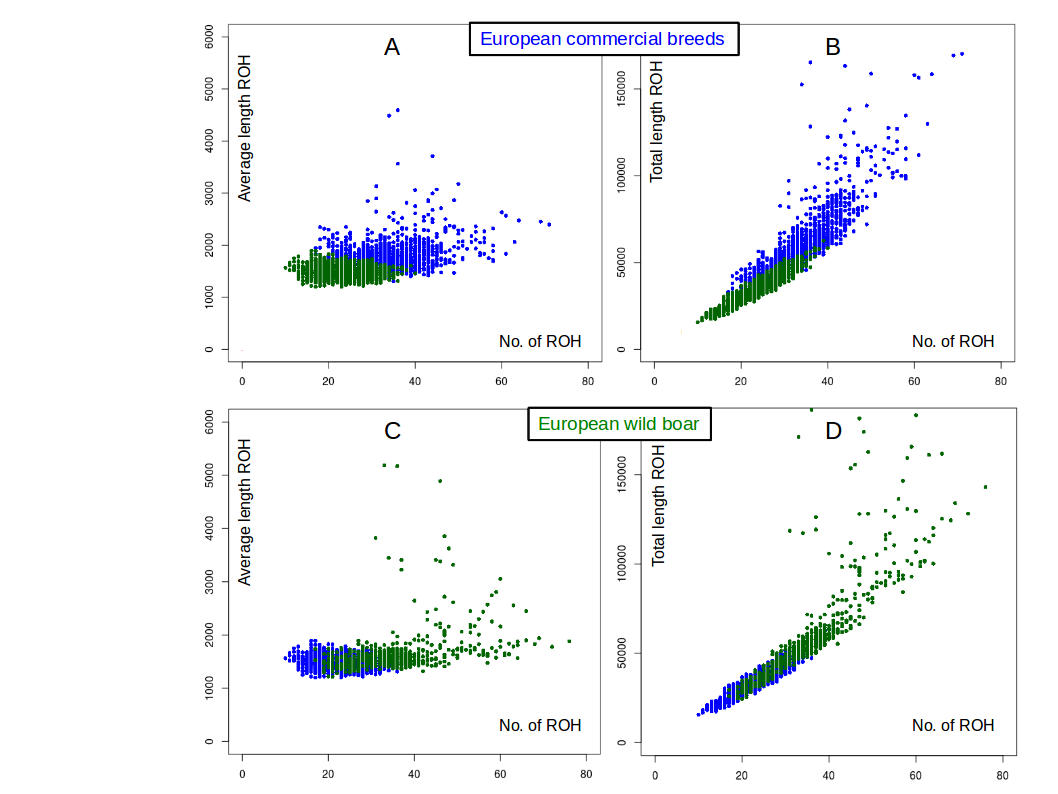

Supplement: Figure S2 — Runs of homozygosity between paired haplotypes. ROHs on chromosome 1 are recorded between pairs of haplotypes that belong to the European wild group (green) or the European commercial group (blue). (A) Number of ROH and average ROH length when a haplotype is paired with a European commercial haplotype. (B) Number of ROH and total ROH length when a haplotype is paired with a European commercial haplotype. (C) Number of ROH and average ROH length when a haplotype is paired with a European wild haplotype. (D) Number of ROH and total ROH length when a haplotype is paired with a European wild haplotype. [file Image2.TIF]
